# Predation of ant species *Lasius alienus* on tick eggs: impacts of egg wax coating and tick species

**DOI:** 10.1038/s41598-022-19300-7

**Published:** 2022-08-30

**Authors:** Sirri Kar, Deniz Sirin, Gurkan Akyildiz, Zafer Sakaci, Sengul Talay, Yilmaz Camlitepe

**Affiliations:** 1 Department of Biology, Tekirdag Namik Kemal University, 59030 Suleymanpasa, Tekirdag Turkey; 2 Department of Basic Health Sciences, Health Sciences Faculty, Marmara University, Istanbul, Turkey; 3 Department of Biology, Balikesir University, Balikesir, Turkey; 4 Department of Biology, Trakya University, Edirne, Turkey

**Keywords:** Behavioural ecology, Animal behaviour, Entomology

## Abstract

Several animal species, including ants, have been reported to be capable of predation on ticks. However, determining factors in most interactions between ticks and predators have not yet been fully deciphered. We hypothesized that the ant species *Lasius alienus*, which is unknown whether it has any impact on ticks, may exhibit predation on the eggs of tick species *Hyalomma marginatum*, *H. excavatum*, and *Rhipicephalus bursa*, and that the tick egg wax can be the main determinant in possible predation. In the study, 6300 tick eggs with the natural wax coating (waxed/untreated) and 2700 dewaxed tick eggs, the wax of which was removed in the laboratory, were repeatedly presented to the foraging workers belonging to three different ant nests in their natural habitat. Depending on the tick species and trials, the rate of the eggs carried by the ants ranged from 12.8 to 52.1% in the waxed and from 59.8 to 78.4% in the dewaxed eggs. It was observed that the dewaxing process both increased the interest of the ants in the eggs and resulted in a reduction in the variation associated with tick species. This study showed that *L. alienus* has a predatory effect on tick eggs, the severity of this impact is closely associated with the tick species, the tick-associated difference is caused by the species-specific property of the egg wax, and the variety in the protective effects of the wax seems to be an evolutional result of the biological and ecological adaptation process of the species.

## Introduction

To date, many kinds of potential predator–prey interactions have been demonstrated between ticks and a wide range of animals, including birds, mammals, and arthropods such as beetles, spiders, and ants^1–3^. Of those, the ants were indicated to be one of the most effective tick predators^4,5^. More than 27 ant species of 17 genera (*Anoplolepis*, *Aphaenogaster*, *Camponotus*, *Crematogaster*, *Ectatomma*, *Formica*, *Iridomyrmex*, *Meranoplus*, *Monomorium*, *Myrmica*, *Notoncus*, *Pheidole*, *Pogonomyrmex*, *Polyrhachis*, *Rhytidoponera*, *Solenopsis*, and *Tapinoma*) have been reported to be effective on different tick species (*Amblyomma* spp., *Boophilus* spp., *Ornithodoros* spp., *Ixodes* spp., *Argas* spp., *Aponomma hydrosauri*, *Rhipicephalus appendiculatus*, *Otobius megnini*, and *Dermacentor variabilis*)^2,6–8^. It is known that ants and other predators can affect ticks in a consumptive or nonconsumptive / behavioral manner and, as a result, may reduce the abundance of ticks in the overlap ranges^8–10^. However, the effects of ants on ticks are closely related to the ant species and the species, developmental stages, and physiological status of the ticks, and as a consequence, the impact of ants on ticks can exhibit fairly high variability^2,8^. Furthermore, there is no sufficient data on the factors determining the tick-ant relationship^11,12^.

Ant predation has been examined in all developmental stages of ticks, but the proportion of the studies based on the eggs is relatively low compared to the other stages^2^. In an egg-based study, the eggs of *O. megnini*, the spinose ear tick, were supplied to five different ant species, and of those, *Tapinoma melanocephalum* was the only species that fed on the eggs^7^. Conflicting results have been reported from the studies^13,14^ carried out to determine the predatory effects of ant species *Pheidole megacephala* on the eggs of *Boophilus* (*Rhipicephalus*) *microplus*^14^. *Rhipicephalus sanguineus* was demonstrated to secrete an acarine allomone when attacked by fire ants, *Solenopsis invicta*^15^. This allomone-based ant deterrence is known to protect ticks from being eliminated within the sympatric range. The eggs, intact and cracked, of tick species *Amblyomma americanum* were not attacked by *S. invicta*, and it was interpreted that this deterrence might be related to the possible presence of the allomone within the eggs^12^.

This study was carried out to determine whether the ant species *Lasius alienus* (Förster, 1850) (Hymenoptera: Formicidae) has any predatory effect on the eggs of tick species *Hyalomma marginatum*, *H. excavatum*, and *Rhipicephalus bursa*, and if the tick egg wax has any protective properties against possible predation. Ticks lay eggs (each 50–100 µg in weight and 0.5–1 mm in length) with a wax coat 0.5–2.0 µm thick, which is secreted by the female-tick-specific glands and organs such as the Gené’s^16,17^. Different molecules have been detected in the wax, such as alkanes, fatty acids, steroids, alcohols, and some specific proteins and lipoproteins^18–20^. However, detailed data on the wax content, especially its bioactive components, are not yet available^20,21^. As for the function of the wax, it has been reported that it reduces water loss, waterproofs the eggs, ensures the proper gas exchange between the eggs and air and holds the eggs together^16,18^. The wax also provides protection against chemical and physical factors such as cold, heat, proteinase K and pronase, or microbial agents including bacteria, fungi, viruses, and protozoa^19–25^.

*Lasius alienus* is one of the most abundant ant species in the Western Palearctic^26^. Its distribution area can range from natural open habitats, light forests, and forest edges to urbanized areas such as wooded residential areas and gardens. The nests can be encountered mostly in the soil, under stones, or other substances, and the nest densities may reach up to 10–50 nests/100 m^2^ in some endemic territories. The number of workers (2­4 mm in length) in a colony can be more than 10,000. In the active periods in hot and warm months, workers establish foraging trails on the ground, in trees, and even in dwellings for food^27,28^. *Lasius alienus* was reported to gather plant nectar, honeydew secreted by aphids and to consume both dead and small living arthropods^28–30^. However, there is no data in the literature on whether this or any other species in the *Lasius* genera have a predatory interaction with ticks. Furthermore, the only definitive association of *L. alienus* with predation on Acarina has been established recently with *Dermanyssus gallinae* (poultry red mite)^31^.

Considering the fact that the workers of *L. alienus* can forage effectively in many different places within the wide range distribution area^27,28,31^, it seems quite possible that several tick species encounter this ant species in their habitat^32^. This ant can feed by scavenging and predating small insects, and it meets their protein needs by hunting large numbers of small invertebrates, especially during larval feeding periods using the central foraging strategy^29^. Whichever invertebrates are abundant in their environment, the ants undoubtedly tend to consume more of them, especially if they are easy to hunt and transport^27–29^. Engorged large female ixodid ticks (around 1–1.5 cm depending on the species) lay a single batch of a large number of eggs (hundreds or thousands depending on the species and feeding levels) for several days or weeks at the hiding points such as cracks, crevices, and spaces under stones or various objects on the ground^21,33^ that the ant can easily reach due to its small size. In ixodid ticks, the female ticks lay eggs at once and die. The next generation continues entirely through the eggs^21^. Referring to these data, it seems hypothetically possible that any level of predation of *L. alienus* on the eggs, which are immobile and easy to reach and carry for the ants, can have a direct effect on the tick community in the overlap ranges. At this point, of course, whether the natural distribution areas of the ant and tick species overlap and, potentially, whether there is a possible evolutionary background between them are of the expected determining factors in a possible predation^34^.

The nests of *L. alienus* can be seen almost everywhere in the soil in its ranging territories, however, the density increases from dry steppe, open pasture and bushlands to cultivated areas, woodlands, and gardens^29^. In this study, three ixodid tick species were selected that are more or less different from each other in terms of biology, ecology, and therefore the probability of encountering *L. alienus* in their natural habitat. Of these, *H. marginatum* is a two-host tick, the immature stages (larvae and nymphs) prefer rabbits, hedgehogs and birds to feed, and the adults (male and female) use particularly cattle as host. The immature stages of mostly three-host tick *H. excavatum*, wild rodents are the preferable host, and a wide range of large wild animals, cattle and some other domestic animals can be the host for the adults. Both species are known as arid environment ticks, however, in accordance with their different host preferences as well, *H. excavatum* is more prevalent in the arid open fields, steppe, and bushlands^32,35^. Both immature and adult stages of two-host tick *R. bursa* use primarily domestic ruminants to feed. Although there is no detailed data on the natural dynamics of *R. bursa*, this species is suspected of having a kind of peri-farm natural dynamics^32^.

## Materials and methods

### Study area and season

**Figure 1:**
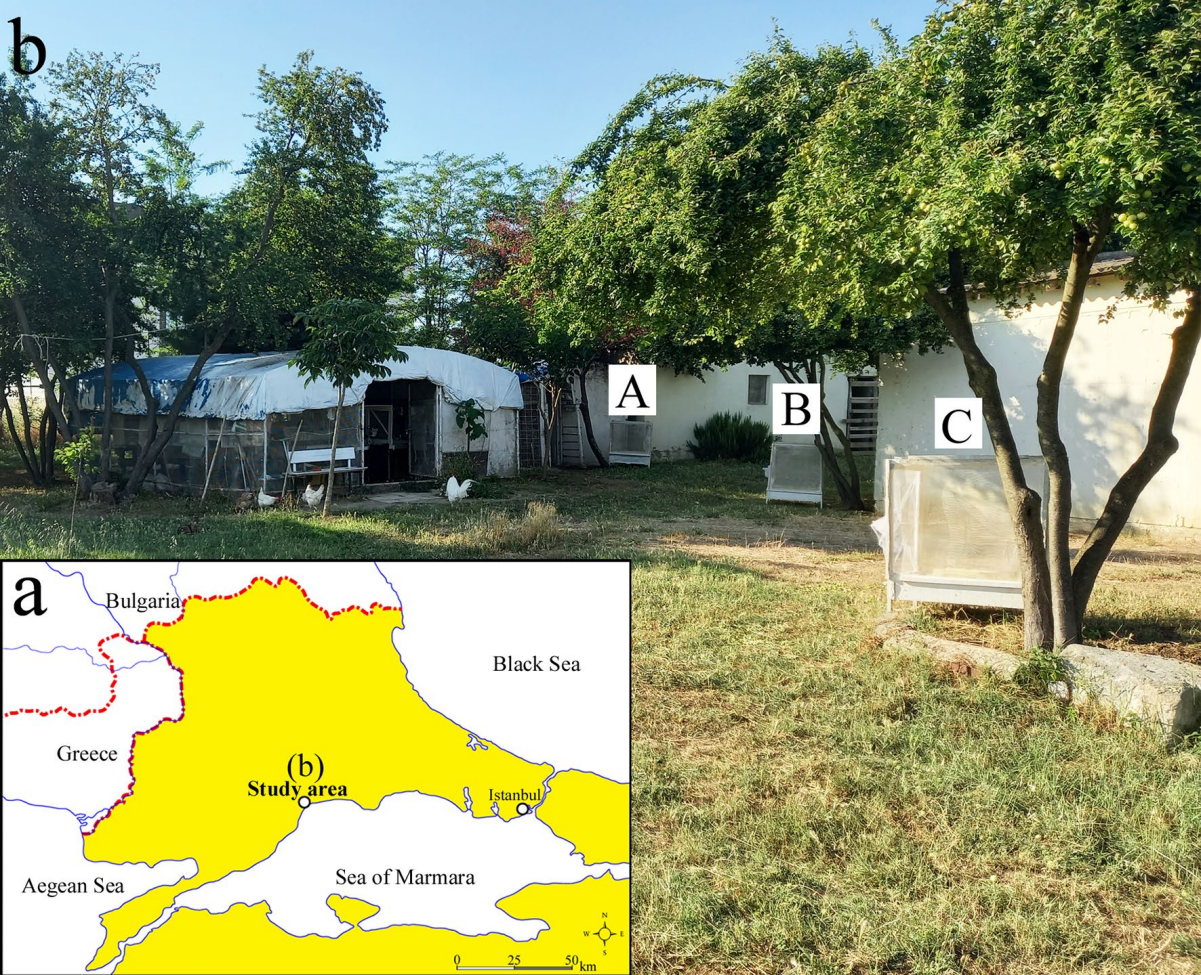
Geographic location (**a**) (retrieved from https://www.simplemappr.net; accessed June 19, 2022) of the study area (**b**) and placement of the cages used in the trials (A, B, and C).

This study was carried out in the field, in the vicinity of Vector Ecology Research Units of our study group, in Turkish Thrace (40° 59′ N, 27° 34′ E; average altitude: 17 m) (Fig. 1a). The trials were performed during the warm season of 2020, between June to September. Selected meteorological parameters of the study year were as follows: the average temperature (min–max) from spring to winter was 13.5 °C (4.1–25.2), 24.5 °C (16.7–32.2), 17.5 °C (8.4–26.8), and 6.7 °C (− 1.9 to 16.6) and mean total rainfall was 109.6 mm, 92.2 mm, 94.2 mm, and 208.9 mm, respectively.

The study area contains greenhouses, gardens, fields, and sparse trees (Fig. 1b). There were ant nests scattered throughout the locality, the species of which were identified as *L. alienus*^26^. The trials were performed on the days without precipitation in the warm months when the activity and population density of the ants were high, in accordance with the well-known seasonal dynamics of the species^27,28^.

### Tick eggs

The eggs belonging to the tick species *Hyalomma marginatum*, *H. excavatum*, and *Rhipicephalus bursa* were used in this study. These tick species can be encountered in the study region, particularly in the rural environment, however in the study location with a peri-urban environment they are not found. In the study area, *Rhipicephalus turanicus* is the only tick species frequently found on the animals, such as cats and dogs, and therefore the only species that the ants in the study area are likely to have encountered naturally.

Adult ticks were fed on New Zealand rabbits (*Oryctolagus cuniculus*). Engorged female ticks were washed with distilled water, dried, put into sterile tubes, and incubated at 25–27 °C and 70–75% relative humidity for egg-laying. The egg batches were monitored daily under a stereomicroscope, and the incubation was continued until the first laid eggs reached the advanced stages of embryogenesis. This point was characterized by the formation of Malpighian tubules as white stripes and the appearance of the rectal sac as a white spot in the eggs. Considering that some physiological and chemical changes can occur in the egg wax during the incubation period^16,18,36^, all the eggs belonging to different periods of laying, and therefore in different periods of the embryonic development stage, were gently mixed, and randomly selected eggs from this mixture were used for predation trials.

In the study, both eggs with the natural wax cover (waxed/untreated) and the eggs that were dewaxed at the laboratory were used. For the dewaxing process, the method described specifically for tick egg wax extraction was used^19^ with some modifications. A chloroform:methanol solution (≥ 99.8%/99–99.4%) was prepared in a 2:1 (v/v) ratio. Ten ml of this solution at room temperature was used for each gram of egg batch. Eggs and the solution were mixed in a glass tube and gently shaken for one minute. Afterward, the eggs were filtered using a stainless strainer, and the same process was repeated three times with distilled water to remove the chemical residue of the solution from the eggs. Since the dewaxed eggs have feeble resistance to drying, they were kept wet at room temperature until used in the trials and presented to the ants on the same day of the dewaxing process. On the days of the trials, waxed eggs were also kept at room temperature and all eggs were prevented from receiving direct sunlight.

### Experimental design and predation trials

For the trials, three cages enclosed by a wire mesh were used (dimensions 70 × 110 cm, h 90 cm, with four legs 15 cm high). The wire mesh cover was perforated at a point (Ø: ~ 5 mm) to allow the regular passage of ants. The cages were placed under three different plum trees about 10 m apart. More than 16 *L. alienus* nests have been identified in an area of 50 m in diameter where cages have been placed. No ant nests belonging to any other species were found within this area, except for two *Messor*^37^ nests located 10 and 16 m from the cage C and one nest of *Cataglyphis aenescens*^37^, which is located 20 m from the cage C. Before the trials, three easily identifiable *L. alienus* nests located next to the trunk of three plum tree which are inspected to have a high number of workers heading for the tree were determined, and the cages were placed next to these nests (Fig. 1b). Although it was not determined whether the nests belong to different colonies or not, it was aimed to ensure that three cages appeal to different nests with this study setup, and the cages were not moved throughout the study.

**Figure 2:**
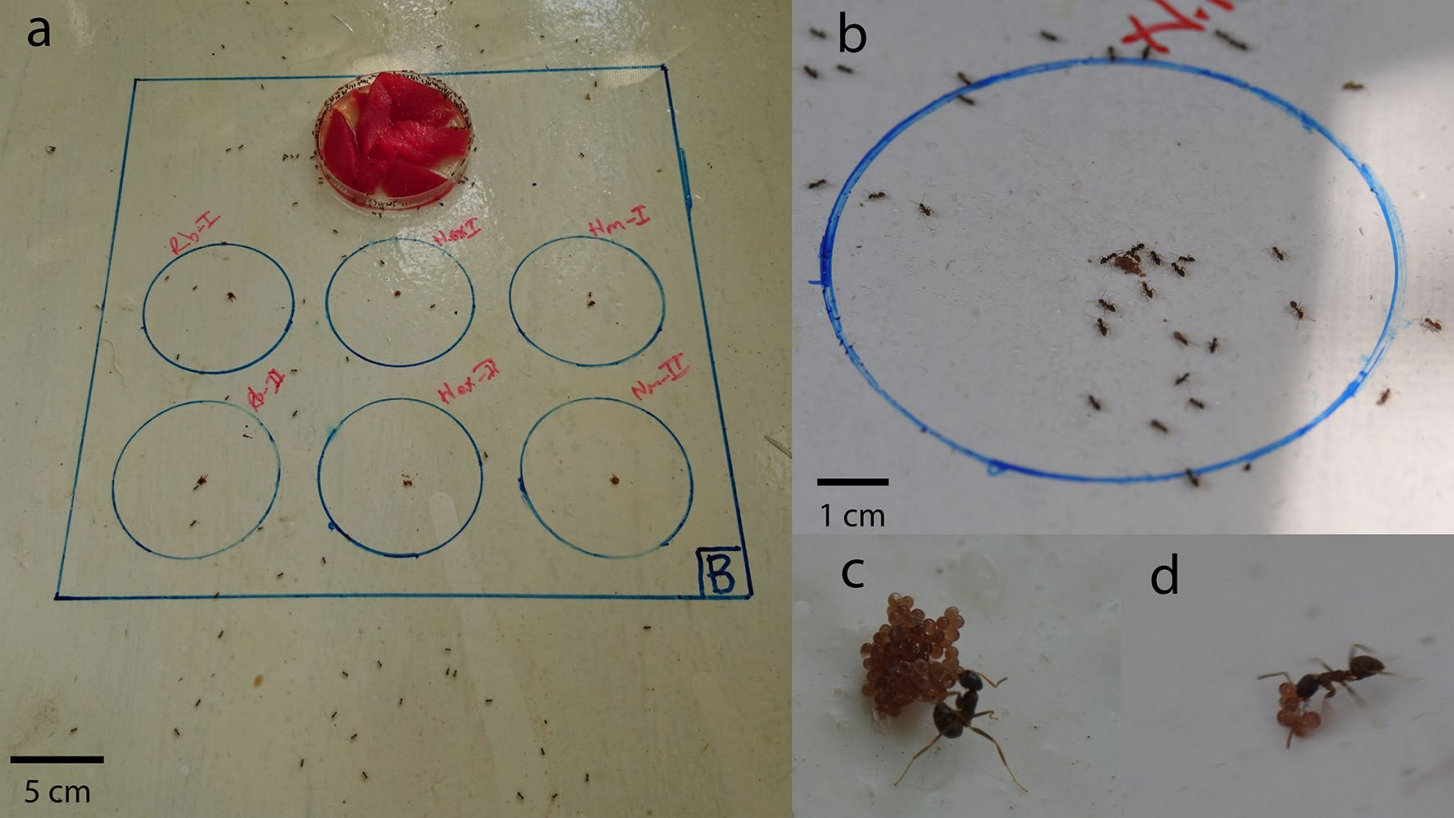
Experimental design in the cages. (**a**) Main square and egg-contained circles drawn on the base of cages, (**b**) an egg-contained circle attacked by the ants, (**c**) an ant exhibiting the behavior of formic acid spraying on the eggs, and (**d**) an ant carrying the tick eggs.

On the white-colored wooden base of each cage, a square (30 × 30 cm) containing six circles (Ø: 8 cm) was drawn. To attract the ants, the petri dish (Ø: 9 cm, depth: 1.5 cm) full of pieces of watermelon and honey (3 ml) was placed on a side of the square (Fig. 2a). To acclimate the ants to the cage environment, supplementation of the watermelon and honey was begun three days before the predation trials, and the contents of dishes were replaced with the fresh ones daily throughout the study.

In the first experiment (Experiment I), 100 eggs of each tick species were placed in the middle of each circle at the base of the cages (Fig. 2a,b) at noon. For each tick species, two circles were allocated in each of the three cages, and as a result, six replications were performed for each tick species in each trial. Considering the particular foraging trails of the ant workers in the cages, the placement of eggs in the circles was randomized in each trial. Following the egg placement in the cages, the behavior of the ants was observed for 1 h continuously. After 24 h, the behavior of the ants was rechecked for 10 min, and the remaining eggs were counted and recorded. This trial was repeated two times more at one-week intervals, using the same three cages standing in the same position. After each trial, the cages were cleaned, the remains of tick eggs were removed and thoroughly wiped with a wet towel, and the articles indicating the tick species were erased to write new ones.

The second (Experiment II) and third experiments (Experiment III) were performed in the same way as Experiment I. Differently, all the eggs used in Experiment II were dewaxed. In Experiment III, waxed and dewaxed eggs were used together, and for each tick species, two circles in each cage were used, one for waxed and the other for dewaxed eggs. Experiments II and III were carried out once using 18 circles at the three cages (six circles for each tick species).

The following method was performed to determine whether the presented eggs affect the number of ants in the cages: Photographs were taken with an interval of 5 min, 1 h before and 1 h after (24 consecutive photos in total for each cage) the eggs were placed in the cages. The number of ants on the base of the cages (at the circles and main square, except for the ones on the watermelon and honey containing Petri dishes) was determined by using the taken photographs.

### Statistical analyzes

The parametric or nonparametric nature of the data was determined by applying Anderson–Darling (for normality) and Levene (homogeneity of variance) tests. The two-tailed Wilcoxon matched pairs signed-ranks tests^38^ were carried out to test the number of ants in the cages before and after the presentation of the tick eggs to the ants, and all the records (belonging to 24 consecutive photos taken with an interval of 5 min, 1 h before and 1 h after the egg supplying to the ants for each cage) recorded during the trials were used for the analysis. To examine whether the interest of the ants in the eggs differed among the eggs (tick species/waxed/dewaxed/mix) in the no-choice bioassay one-way analysis of variance (ANOVA) was used, followed by Tukey’s post-hoc HSD test^39^. Kruskal–Wallis tests were applied to compare more than two independent samples and Mann–Whitney U tests for comparison of two independent samples^38^. We used the Kruskal–Wallis test to examine whether there were differences between the cages regarding the counts of eggs carried by the ants. Subsequently, we used Mann–Whitney U tests to examine the cages for differences. To avoid inflation of the first type of error due to multiple testing, we applied Bonferroni–Holm^40^ correction for each comparison. A value of *P* < 0.05 was accepted as significant. Kolmogorov–Smirnov, Levene, Kruskal–Wallis, and Mann–Whitney U tests were performed using SPSS version 15.0 (SPSS Inc., Chicago, IL, USA).

### Ethical approval

For this type of study formal consent is not required.

## Results

**Figure 3:**
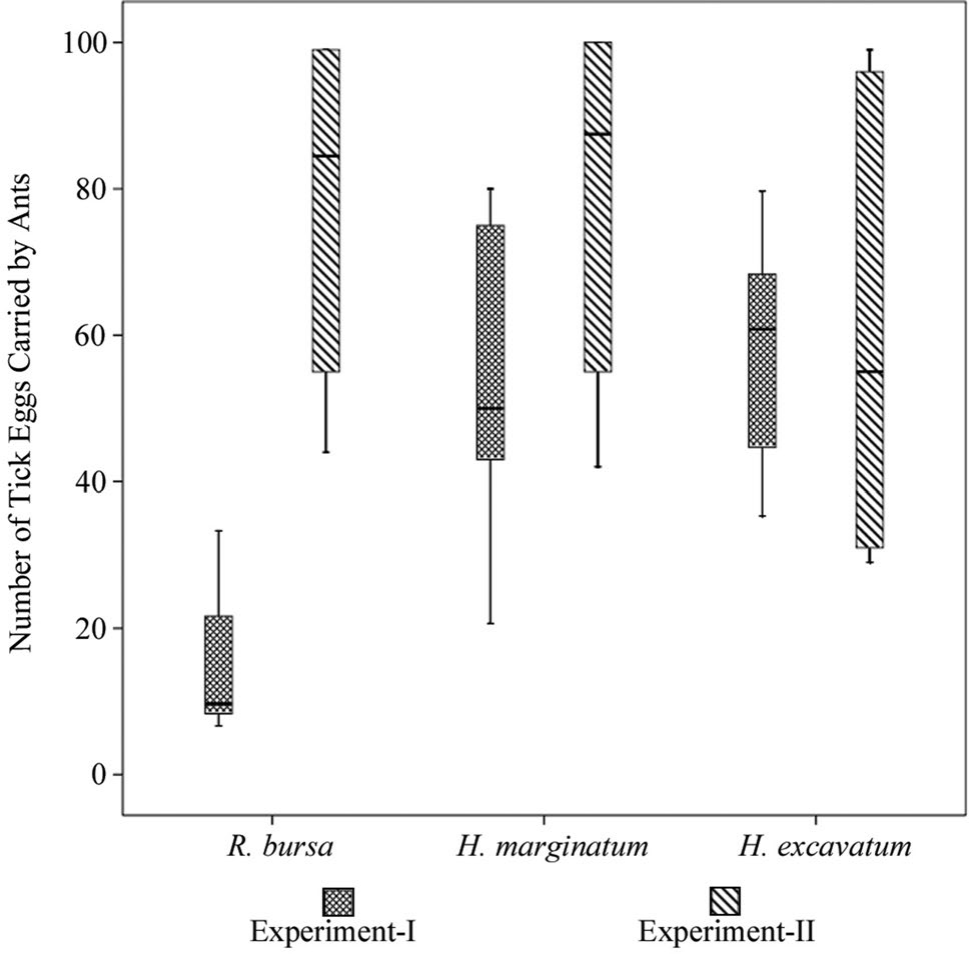
The number eggs with wax coating (Experiment I) and dewaxed (Experiment II) of the tick species carried by the ants.

Throughout the study, a total of 9000 tick eggs, 6300 waxed and 2700 dewaxed, were presented to the three different ant colonies. Of those, 2385 (37.9%) waxed and 1857 (68.8%) dewaxed eggs were carried by the ants (Fig. 2b,d). For each tick species, 2100 waxed and 900 dewaxed eggs were used in the trials. The numbers of waxed and dewaxed eggs carried from *R. bursa* (Rb) were 268 (12.8%) and 613 (68.2%), from *H. marginatum* (Hm) 1024 (48.8%) and 705 (78.4%), and from *H. excavatum* (He) 1093 (52.1%) and 538 (59.8%), respectively (Tables 1, 2). One-way ANOVA analyzes revealed a significant difference (F: 6.561 and sig.: 0.000) in the number of eggs carried by the ants, both between the tick species and between the waxed and dewaxed eggs. According to Post-Hoc (Tukey HSD) test, *R. bursa* exhibits a statistically significant intraspecies variation in waxed eggs (Rb–He, *P* = 0.023) and in waxed–dewaxed eggs comparations (Rb–Rb, *P* = 0.000, Rb–He *P* = 0.014, and Rb–Hm *P* = 0.000). However, there was no significant variation between the species for dewaxed eggs comparations (Fig. 3).

**Table 1:** The number of the waxed tick eggs carried by the ants within 24 h in Experiment I.

Tick species	No of			Number of eggs		The average number of eggs carried from each circle (range)
	Trial	Cage	Circle	Presented	Carried	
*R. bursa*	1	A	1	100	0	14.9 (0–100)
			2		29	
		B	1		0	
			2		0	
		C	1		0	
			2		0	
	2	A	1		29	
			2		0	
		B	1		0	
			2		0	
		C	1		65	
			2		100	
	3	A	1		0	
			2		0	
		B	1		20	
			2		25	
		C	1		0	
			2		0	
*H. marginatum*	1	A	3	100	67	53.1 (0–100)
			4		33	
		B	3		33	
			4		29	
		C	3		100	
			4		100	
	2	A	3		37	
			4		29	
		B	3		100	
			4		96	
		C	3		100	
			4		100	
	3	A	3		25	
			4		0	
		B	3		22	
			4		20	
		C	3		40	
			4		25	
*H. excavatum*	1	A	5	100	100	58.3 (0–100)
			6		100	
		B	5		79	
			6		6	
		C	5		97	
			6		100	
	2	A	5		100	
			6		6	
		B	5		100	
			6		100	
		C	5		10	
			6		100	
	3	A	5		39	
			6		0	
		B	5		20	
			6		60	
		C	5		27	
			6		5

**Table 2:** The number of the waxed and dewaxed tick eggs carried by the ants within 24 h in Experiment II and III.

Tick species	Egg type	No of		Number of eggs		The average number of eggs carried from each circle (range)
		Cage	Circle	Presented	Carried	
**Experiment II**						
*R. bursa*	Dewaxed	A	1	100	81	77.7 (44–99)
			2		44	
		B	1		88	
			2		55	
		C	1		99	
			2		99	
*H. marginatum*		A	3	100	42	78.7 (42–100)
			4		55	
		B	3		77	
			4		98	
		C	3		100	
			4		100	
*H. excavatum*		A	5	100	29	60.8 (29–99)
			6		31	
		B	5		53	
			6		57	
		C	5		99	
			6		96	
**Experiment III**						
*R. bursa*	Dewaxed	A	1	100	17	49.0 (17–97)
		B	1		33	
		C	1		97	
	Waxed	A	2	100	0	0 (0–0)
		B	2		0	
		C	2		0	
*H. marginatum*	Dewaxed	A	3	100	56	78.0 (56–100)
		B	3		100	
		C	3		78	
	Waxed	A	4	100	0	22.7 (0–43)
		B	4		43	
		C	4		25	
*H. excavatum*	Dewaxed	A	5	100	27	57.7 (27–84)
		B	5		84	
		C	5		62	
	Waxed	A	6	100	0	14.7 (0–44)
		B	6		0	
		C	6		44

Regarding the behaviors of the ants, it was observed that the ants had a high interest in the eggs in the 1-h period immediately after the eggs were presented, and a certain number of the eggs were carried by the ants within a day. However, in the observations at the 24th hour, it was inspected that the interest in all eggs had disappeared. The same behavior pattern was obtained in the trials conducted at one-week intervals in the same cages kept in the same place.

The number of ants in the areas on the bottoms of the cages during the 1-h period just before and after the egg-supplying varied depending on the tick species and whether the eggs were waxed or dewaxed. While an increase was recorded in the number of ants between 5.7 and 132.1% in all groups, there was a decrease (− 62.5%) in the circle containing the waxed *H. excavatum* eggs in Experiment III in which the waxed and dewaxed eggs were supplied together in the same cages. These arithmetic values were also proven by the Wilcoxon Signed Rank test. This test showed that all the variations were statistically significant at various levels except for the total number of ants in the main square in the cages in Experiment III (Table 3).

**Table 3:** The number of the ants in the cages at any time during the 1-h period before and after the egg-supplying.

The area on the floor of the cage	Egg type	Mean number of ants in the cage at any time in 1 h period (range)		Proportional change in the number of ants following the egg-supplying (*P* values according to the Wilcoxon Signed Rank test)
		Before the egg-supplying	After the egg-supplying	
**Experiment I**				
*R. bursa* circle	Waxed	1.3 (0–5)	2.0 (0–7)	+ 53.8% (**< 0.000**)
*H. marginatum* circle		0.7 (0–3)	0.9 (0–5)	+ 34.7% (**0.007**)
*H. excavatum* circle		1.0 (0–6)	1.9 (0–6)	+ 77.4% (**< 0.000**)
Whole square		42.5 (22–60)	47.4 (30–72)	+ 11.6% (**0.001**)
**Experiment II**				
*R. bursa* circle	Dewaxed	2.0 (0–9)	3.0 (0–11)	+ 50.0% (**< 0.000**)
*H. marginatum* circle		1.4 (0–8)	2.4 (0–12)	+ 75.5% (**< 0.000**)
*H. excavatum* circle		0.7 (0–7)	1.7 (0–7)	+ 132.1% (**< 0.000**)
Whole square		52.6 (27–78)	59.3 (41–78)	+ 12.7% (**0.021**)
**Experiment III**				
*R. bursa* circle	Waxed	0.3 (0–3)	0.4 (0–2)	+ 50.0% (**0.000**)
	Dewaxed	1.6 (0–5)	2.4 (0–9)	+ 42.4% (**< 0.028**)
*H. marginatum* circle	Waxed	1.5 (0–6)	2.0 (0–7)	+ 35.2% (**0.007**)
	Dewaxed	4 (0–13)	5.9 (0–17)	+ 47.3% (**< 0.000**)
*H. excavatum* circle	Waxed	0.3 (0–1)	0.1 (0–1)	− 62.5% (**0.025**)
	Dewaxed	3.4 (0–9)	4.2 (0–8)	+ 23.4% (**0.005**)
Whole square		85.8 (36–166)	90.7 (56–126)	+ 5.7% (0.155)

Significant values are in [bold].

When all the results of trials in the study were evaluated together, the average number of the ants in the whole square on the bottom of the cage at any time during the 1-h period before and after the egg-supplying was 69.8 and 68.5 at the cage A, 42.0 and 52.1 at the cage B, and 54.4 and 61.7 at the cage C, respectively. In total, the number of eggs carried by ants was 976, 1398 and 1868 at cages A, B, and C, respectively. Kruskal–Wallis test showed that there is a statistically significant difference between the cages in terms of the ant numbers before (*χ*^2^ = 19.160; df = 2; *P* < 0.000) and after (*χ*^2^ = 8.082, df = 2, *P* = 0.018) egg-supplying, and the egg numbers carried by ants (*χ*^2^ = 6.912, df = 2, *P* = 0.032). According to Mann–Whitney *U*-test, the results showed that a statistically significant difference between cages depends on the counts of the ants before the egg-supplying (A–B, Z =  − 4.172, *P* < 0.000; B–C, Z =  − 4.092, *P* = 0.002;), after the egg-supplying (A–B, Z =  − 2.766, *P* = 0.006; B–C, Z =  − 2.137, *P* = 0.033), and the egg numbers carried by ants ((A–C, Z =  − 2.466, *P* = 0.014). However, Mann–Whitney *U*-test revealed that the difference in the number of ants in the cages may not always be directly related to the number of eggs carried by the ants.

## Discussion

This study revealed that the ant species *L. alienus* shows certain interest levels in the eggs of the tick species *H. marginatum*, *H. excavatum*, and *R. bursa*. *Lasius alienus* workers are known to gather sweets for their energy needs and also collect both dead and live insects for protein requirements of the larvae, queen, and workers^28–30^. Given these feeding characteristics, it is not surprising behavior that this ant can also benefit from tick eggs as an alternative source of protein. During the trials, it was observed that the ants occasionally exhibited formic acid spraying behavior on the eggs (Fig. 2c). In fact, formic acid, which can be lethal for tick immatures^41^, is used by ants to immobilize live prey^31^. The reason or purpose of the acid spraying behavior on the immobile eggs in our trials could not be justified.

The level of interest of *L. alienus* in the eggs was closely related to the tick species, and the results revealed that the eggs of *Hyalomma* species, particularly *H. excavatum*, were preferred more by the ant compared to the eggs of *R. bursa*. Similar variability has been clearly demonstrated previously regarding different species of ticks and ants^7,13,14^. However, presenting the eggs to the ants after the dewaxing process resulted in both more significant interest in the eggs and a decrease in the differences between the eggs of the tick species. This result indicates that the wax coating of eggs in some tick species such as *R. bursa* can be one of the most crucial barriers protecting the eggs from the predation of *L. alienus*. Although the antimicrobial activity of the wax has been well documented^22–25^, as far as we know, our result provides the first accurate evidence for its anti-predatory efficacy. Furthermore, the anti-predatory activity of the egg wax of *R. bursa* against *L. alienus* was clearly more substantial than the egg wax of *H. marginatum* and particularly the egg wax of *H. excavatum*. The wax’s chemical properties and antimicrobial efficacy can vary dramatically depending on the species of the agents and ticks^16,42^. In line with this fact, a recent study carried out by our study group showed that the egg wax of *R. bursa* is effective against *Candida tropicalis* at certain levels, but the wax of *H. marginatum* is not^25^.

The divergences in the egg-laying sites and habitat preferences of *H. marginatum*, *H. excavatum*, and *R. bursa* may, of course, be the reason for the difference in their egg wax activities, as previously reported in these^25^ and some other tick species^16,42^. However, biological differences between the species also seem to play a role in species-specific wax differences, at least in its antipredatory activity. The adults of the *Hyalomma* species feed in the spring and summer. The entire process of egg-laying, larval hatching, and attachment of the larvae to the host can be completed within a few weeks under field conditions in Turkey. Although the adults of *R. bursa* feed in hot months (primarily around June), the egg-laying and larval hatching process can take several weeks, and the immature stages are found on the host in the late autumn or winter^25,32^. This information indicates that the more protective feature of egg wax of *R. bursa* is most likely the natural consequence of an evolutionary adaptation, possibly driven by prolonged exposure of the eggs to environmental factors. Given that tick eggs can be exposed to a plethora of biological or nonbiological factors in their natural environment, it seems more plausible that the acquired properties such as featured wax could be used by ticks as non-specific or broad-spectrum protection. Of course, it is difficult to predict the impact of *L. alienus* or other ant species in this evolutional background. However, some biological and ecological facts about the ant and tick species seem to indicate such a possibility as well. It is known that the density of *L. alienus* increases from dry open pasture and bushlands to cultivated areas, woodlands, and gardens^29^. There is no detailed data about the spatial distribution patterns of the tick species used in this study. However, depending on their biology, ecology, and host preferences, it can be accepted that the density of *R. bursa* increases in the same direction as *L. alienus*, that of *H. excavatum* decreases, and that of *H. marginatum* follows a course possibly between these two tick species^32,35^. The fact that the waxed eggs of *R. bursa*, which is most likely to encounter *L. alienus* in the overlapping ranges, are more likely resistant against the predation compared to the eggs of *H. excavatum*, which is least likely to encounter this ant, may indicate a possible co-evolution.

In a recent study, *L. alienus* was observed to be an effective predator on all stages of poultry red mite *Dermanyssus gallinae*, including the eggs and even the eggshells^31^. Poultry red mite is a small (adults, ~ 1 mm in length), nocturnally active, temporary ectoparasite, which inhabits relatively sheltered indoors or nests and mostly hides in narrow spaces during the day^43^. Given this mite’s natural lifecycle and habitat, it is not surprising that it has not developed a remarkably effective protection mechanism against the diurnally active and relatively larger predators such as *L. alienus* (workers, 2­4 mm in length). Although an engorged female tick can also adjust the time and niche of the detachment from the host^32,33^, this is likely related to an evolutionary process to ensure that its larvae can reach their host. Depending on the species, engorged female ticks hide in sheltered cracks or crevices on the ground and lay eggs in a batch at the hiding point^33^. Still, due to their large size and poor mobility, they take shelter mostly within a few meters of the detachment area^33^. The hiding place of engorged ticks is mostly wide enough for ants or smaller potential predators to easily reach. Possible perpetual exposure of the eggs to the multifarious environmental factors seems to be one of the potential reasons why ticks have a higher resistance to the ants than *D. gallinae*, as is the case in many co-evolution-based predator–prey relationships in nature^43^.

It was observed that the ants exhibited greater foraging intensity at the beginning of the trials, however, this interest disappeared within 24 h. The same daily alteration of the interest was repeated in the trials conducted with the same ant nest at one-week intervals. As the reason for this daily alteration, it is possible to speculate some potential justifications, such as (i) the amount of protein supply that a certain *L. alienus* nest can carry over a certain period is limited^29^, (ii) the ants may prefer to feed on different sources instead of a single source of protein/amino acids^29^, (iii) the deterrent or detrimental effect of the egg wax may be limiting the number of eggs that can be consumed within a certain time frame, and (iv) these and many other possible factors may be affecting together. Detailed studies are needed to reveal specific reason or reasons for this behavior of the ants.

The results indicate that *L. alienus* can theoretically be assumed to have a possible elimination potential over *H. excavatum*, *H. marginatum*, and even *R. bursa* within their sympatric range. On the other hand, our results also showed that the repulsive effect of the tick egg wax might be an important barrier to the predation of *L. alienus* and related elimination. However, although the number of waxed eggs that a particular ant nest can consume in a certain period is limited, workers belonging to neighboring colonies should also be considered. This time, the fact that ant colonies tend to defend their range area against other colonies^28^ emerges as a possible factor that may protect the tick eggs from an excessive pressure of the predation of the ant community in the vicinity. In ticks, such resistance to ant-mediated elimination was also demonstrated in relation to the red imported fire ant *Solenopsis invicta*. Although this ant species can reduce the population of some tick species in its range territories in the Americas, it is unable to eradicate them, and in this failure, some defense strategies possessed by ticks (e.g., masking with allomone secretion against the ants) was suggested to play a crucial role^12^.

Ticks are mostly resistant to various environmental drivers^21^. In fact, a certain level of suppression of ticks by predators such as ants may even be beneficial for the balanced maintenance of such a parasite in nature, which has excessive reproductive potential^32,33^. It is known that an uncontrolled increase in the population density of a tick species in a given territory can cause significant damage to the hosts may critically reduce their density^11^ or can force them to a displacement^44,45^ due to direct harmful effects or tick-borne infectious diseases. As with many other host-parasite-agent or prey-predator relationships in nature^46–48^, inevitably, this can negatively affect ticks through the trophic cascade, most of which are specific to one or only a few host species^32,33,43^. Furthermore, it is known that some tick-borne disease agents may also disrupt or change some biological/physiological processes in ticks^21^. Investigating whether any tick-borne agent causes a change in the content of egg wax, or its anti-predator efficiency seems worthy of investigation as it may provide a better understanding of the role of infectious agents and predators in the natural dynamics of ticks and tick-borne diseases.

## Conclusions

The results of this study revealed that, (i) *Lasius alienus* can exhibit predation on the eggs of the tick species *H. marginatum*, *H. excavatum*, and *R. bursa*, indicating a potential bio-suppression on these and possibly some other tick species in nature, (ii) the level of the predation varies depending on the tick species, and this circumstance is directly related to the species-specific protective capacity of the egg wax, and (iii) considering the other known properties, tick egg wax seems worthwhile to research to reveal its bioactive substances and their effects that may be benefited for different purposes. Hence, what can be concluded is that this area shows much promise and further investigation into this phenomenon is needed.

## Data Availability

All data generated or analysed during this study are included in this published article.

## References

[1] Mwangi EN, Dipeolu OO, Newson RM, Kaaya G, Hassan SM (1991). Predators, parasitoids and pathogens of ticks: A review. Biocontrol Sci. Technol..

[2] Samish M, Alekseev E (2001). Arthropods as predators of ticks (Ixodoidea). J. Med. Entomol..

[3] Fischhof IR, Burtis JC, Keesing F, Ostfeld RS (2018). Tritrophic interactions between a fungal pathogen, a spider predator, and the blacklegged tick. Ecol. Evol..

[4] Barré N, Mauleon H, Garris GI, Kermarrec A (1991). Predators of the tick *Amblyomma variegatum* (Acari: Ixodidae) in Guadeloupe, French West Indies. Exp. Appl. Acarol..

[5] Samish M, Ginsberg HS, Glazer I (2004). Biological control of ticks. Parasitology.

[6] Samish M, Rehacek J (1999). Pathogens and predators of ticks and their potential in biological control. Annu. Rev. Entomol..

[7] Diyes GCP, Karunarathna NB, Silva THSE, Karunaratne WAIP, Rajakarunab RS (2017). Ants as predators of the spinose ear tick, *Otobius megnini* (Dugés) in Sri Lanka. Acarologia.

[8] Guarnieri LD, McBride SE, Groden E, Gardner AM (2021). Interactions between sympatric invasive European fire ants (*Myrmica rubra*) and blacklegged ticks (*Ixodes scapularis*). PLoS ONE.

[9] Burtis JC, Pflueger C (2017). Interactions between soil-dwelling arthropod predators and *Ixodes scapularis* under laboratory and field conditions. Ecosphere.

[10] Kjeldgaard MK, Takano OM, Bockoven AA, Teel PD, Light JE, Hamer SA, Hamer GL, Eubanks MD (2019). Red imported fire ant (*Solenopsis invicta*) aggression influences the behavior of three hard tick species. Exp. Appl. Acarol..

[11] Burtis JC, Yavitt JB, Fahey TJ, Ostfeld RS (2019). Ticks as soil-dwelling arthropods: An intersection between disease and soil ecology. J. Med. Entomol..

[12] Showler AT, Osbrink WLA, Dorsey BN, Caesar RM (2019). Metastriate ixodid life stages protected from predatory ants in Texas. Environ. Entomol..

[13] De la Vega R, Diaz G, Palacios ME (1984). *Pheidole megacephala* as a predator of *Boophilus microplus*, qualitative and quantitative aspects. Rev. Salud Anim..

[14] Castineiras A, Jimeno G, Lopez M, Sosa LM (1987). Effect of *Beauveria bassiana*, *Metarhizium anisopliae* (Fungi: Imperfecti) and *Pheidole megacephala* (Hymenopthera: Formicidae) on eggs of *Boophilus microplus* (Acarina: Ixodidae). Rev. Salud Anim..

[15] Yoder JA, Benoit JB, Bundy MR, Hedges BZ, Gribbins KM (2009). Functional morphology of secretion by the large wax glands (sensilla sagittiformia) involved in tick defense. Psyche (Camb Mass).

[16] Lees AD, Beament JW (1948). An egg-waxing organ in ticks. Q. J. Microsc. Sci..

[17] Labruna MB, Leite RC, Oliveira PR (1997). Study of the weight of eggs from six ixodid species from Brazil. Mem. Inst. Oswaldo Cruz..

[18] Booth TF (1992). Observation on the composition and biosynthesis of egg wax lipids in the cattle tick, *Boophilus microplus*. Exp. Appl. Acarol..

[19] Arrieta MC, Leskin BK, Kaufman WR (2006). Antimicrobial activity in the egg wax of the African cattle tick *Amblyomma hebraeum* (Acari: Ixodidae). Exp. Appl. Acarol..

[20] Yu Z, Thomson ELS, Liu J, Dennis JJ, Jacobs RL, Kaufman WR (2012). Antimicrobial activity in the egg wax of the tick *Amblyomma hebraeum* (Acari: Ixodidae) is associated with free fatty acids C16:1 and C18:2. Exp. Appl. Acarol..

[21] Sonenshine DE, Roe RM (2014). Biology of Ticks.

[22] Lima-Netto S, Pinheiro A, Nakano E, Mendonca RMZ, Barros-Battesti DM, Mendonca RZ (2012). Antiviral effect of the egg wax of *Amblyomma cajennense* (Acari: Ixodidae). Cytotechnology.

[23] Alduini N, Silva M, Franzolin M, Mendonca R, Lima-Netto S (2014). Antimicrobial activity from ticks eggs waxes. BMC Proc..

[24] Yang X, Jia Q, Chen J, Hou Y, Zhai S, Yu Z, Liu J (2017). Antibacterial activity of eggs and egg wax covering of selected Ixodid (Acari: Ixodidae) ticks. J. Entomol. Sci..

[25] Bilgin N, Hacioglu M, Bozkurt Guzel C, Erdal B, Kar S (2020). In vitro anticandidial efficacy of tick egg wax from *Hyalomma marginatum*, *Rhipicephalus bursa* and *Dermacentor marginatus*. Clin. Exp. Health Sci..

[26] Seifert B (2020). A taxonomic revision of the Palaearctic members of the subgenus *Lasius* s. str. (Hymenoptera, Formicidae). Soil Org..

[27] Robinson WH (2005). Handbook of Urban Insects and Arachnids.

[28] Seifert B (2018). The Ants of Central and North Europe.

[29] Collingwood CA (1979). The formicidae (Hymenoptera) of Fennoscandia and Denmark. Fauna Entomol. Scand..

[30] Dussutour A, Simpson SJ (2009). Communal nutrition in ants. Curr. Biol..

[31] Kar S, Akyildiz G, Sirin D, Rodriguez SE, Camlitepe Y (2021). First evidence of predation of the ant species *Lasius alienus* on the poultry red mite *Dermanyssus gallinae*. Acarologia.

[32] Kar S, Akyildiz G, Guven E, Bente D, Vatansever Z (2021). Monthly infestation characteristics of ticks on cattle in Thrace, a Crimean Congo hemorrhagic fever-endemic area of Turkey. Parasitol. Res..

[33] Akyildiz G, Guven E, Tufek H, Bente D, Vatansever Z, Kar S (2021). Monthly dynamics of the cold-adapted one-host biological north form of *Hyalomma scupense* under the influence of the warm summer subtype of the Mediterranean climate in Turkey. Parasitol. Int..

[34] van Velzen E, Gaedke U (2017). Disentangling eco-evolutionary dynamics of predator–prey coevolution: The case of antiphase cycles. Sci. Rep..

[35] Kar S, Gargili Keles A, Nuttall P (2021). Passible direct and human-mediated impact of climate change on tick populations in Turkey. Climate, Ticks and Disease.

[36] Sonenshine DE, Tigner JA (1969). Oviposition and hatching in two species of ticks in relation to moisture deficit. Ann. Entomol. Soc. Am..

[37] Agosti D, Collingwood CA (1987). A provisional list of the Balkan ants (Hym. Formicidae) with a key to the worker caste. II. Key to the worker caste, including the European species without the Iberian. Mitt. Schweiz. Entomol. Ges..

[38] Steel RGD, Torrie JH (1960). Principles and Procedures of Statistics, With Special References to the Biological Sciences.

[39] Kalayci S (2005). SPSS Applied Multivariate Statistical Techniques.

[40] Holm S (1979). A simple sequentially rejective multiple test procedure. Scand. J. Stat..

[41] Showler AT, Dorsey BN, Caesar RM (2020). Effects of formic acid on *Amblyomma americanum* (Ixodida: Ixodidae) larvae and nymphs. J. Med. Entomol..

[42] Teel PD (1984). Effect of saturation deficit on eggs of *Boophilus annulatus* and *B. microplus* (Acari: Ixodidae). Ann. Entomol. Soc. Am..

[43] Roy L, Chauve C, Sabelis MW, Bruin J (2010). The genus *Dermanyssus* (Mesostigmata: Dermanyssidae): History and species characterization. Trends in Acarology: Proceedings of the 12th International Congress.

[44] Lang JM, Benbow ME (2013). Species interactions and competition. Nat. Educ. Knowl..

[45] Fritzsche A, Allan BF (2012). The ecology of fear: host foraging behavior varies with the spatio-temporal abundance of a dominant ectoparasite. EcoHealth.

[46] Moore SM, Borer ET, Hosseini PR (2010). Predators indirectly control vectorborne disease: linking predator–prey and host–pathogen models. J. R. Soc. Interface.

[47] Buck JC, Ripple WJ (2017). Infectious agents trigger trophic cascades. Trends Ecol. Evol..

[48] Winnie J, Creel S (2017). The many effects of carnivores on their prey and their implications for trophic cascades, and ecosystem structure and function. Food Webs.

